# Antenatal Iron Supplementation Regimens for Pregnant Women in Rural Vietnam and Subsequent Haemoglobin Concentration and Anaemia among Their Infants

**DOI:** 10.1371/journal.pone.0125740

**Published:** 2015-04-30

**Authors:** Thach Duc Tran, Jane Fisher, Sarah Hanieh, Tuan Tran, Julie Anne Simpson, Ha Tran, Beverley-Ann Biggs

**Affiliations:** 1 Research and Training Centre for Community Development, Hanoi, Vietnam; 2 Jean Hailes Research Unit, School of Public Health and Preventive Medicine, Monash University, Melbourne, Victoria, Australia; 3 Department of Medicine (RMH/WH), The University of Melbourne, The Royal Melbourne Hospital, Melbourne, Victoria, Australia; 4 Centre for Molecular, Environmental, Genetic & Analytic Epidemiology, Melbourne School of Population and Global Health, The University of Melbourne, Melbourne, Victoria, Australia; University of São Paulo, BRAZIL

## Abstract

**Background:**

Little evidence about the effects of antenatal iron supplementation on infant anaemia is available. The aim was to compare effects on six-month-old infants’ Haemoglobin (Hb) concentration and anaemia of daily iron–folic acid (IFA), twice-weekly IFA with or without other micronutrients (MMN) and usual antenatal care in rural Vietnam.

**Methods and Findings:**

Secondary data analysis from: a prospective population-based observational study (OS) which examined effects of antenatal psychosocial factors, anaemia and iron deficiency on infant development and health; and a three-arm cluster randomised trial (CRT) of different antenatal iron supplementation regimens. In the OS 497 women (<20 weeks gestation) from 50 randomly-selected communes participated, and in the CRT 1,258 pregnant women (<16 weeks gestation) in 104 communes were allocated randomly to trial arms. The main outcome was six-month-old infant Hb concentration. Baseline data included women’s socio-demographic characteristics, reproductive health, Hb and serum ferritin. Mean differences in infant Hb and odds ratios of infant anaemia between CRT arms and OS were calculated by multivariable regression models, controlling for baseline differences and clustering, using robust standard errors.

Infant anaemia prevalence was 68.6% in the OS, 47.2% daily IFA, 53.5% weekly IFA, and 50.3% MMN conditions. After adjustment, mean infant haemoglobin levels in daily IFA (mean difference = 0.95 g/dL; 95%CI 0.7-11.18); weekly IFA (0.91; 95%CI 0.69-1.12) and MMN (1.04; 95%CI 0.8-1.27) were higher than in the OS. After adjustment there were lower odds ratios of anaemia among infants in the daily IFA (OR = 0.31; 95% CI 0.22-0.43), weekly IFA (0.38; 95%CI 0.26-0.54) and MMN (0.33; 95%CI 0.23-0.48) groups than in the OS.

**Conclusions:**

Infant anaemia is a public health problem in Vietnam and other resource-constrained countries. All supplementation regimens could have clinically significant benefits for Hb and reduce anaemia risk among six-month-old infants. Universal provision of free intermittent iron supplements is warranted.

## Introduction

Anaemia, often caused by iron deficiency, is highly prevalent among women and children in low- and middle-income countries (LAMIC) [1]. The World Health Organization (WHO) recommends that pregnant women take antenatal iron-folic acid supplements (IFA, daily dose of 60 mg of elemental iron and 400 μg of folic acid starting as soon as possible after gestation begins [2]) to maintain robust iron stores and prevent the development of anaemia. Although, daily IFA supplementation has been the standard regimen for decades, WHO has recently recommended the use of intermittent IFA in non-anaemic pregnant women because it has fewer side effects and compliance is increased [3]. In resource-constrained settings, deficiencies in micronutrients such as vitamins A, B6, B12, C and riboflavin and zinc are also common among pregnant women and likely to coexist with iron deficiency [4]. Multiple micronutrient supplementation (MMN) during pregnancy is emerging as an approach to improve maternal and infant health in the setting of poverty [5].

There is an existing body of evidence on the effects of maternal antenatal folate status and iron level, antenatal IFA and MMN supplementation on pregnancy outcomes and infant development and health in LAMIC [2,6–10]. Nevertheless, there is a lack of studies examining the effect of these antenatal supplementations on infant anaemia [2], an important outcome that could affect infant development and health later in life [11,12]. To date there is only one randomised-controlled trial conducted by Preziosi et al. in Niger [13] that has reported the effect of antenatal iron supplementation on infants’ haemoglobin concentration (Hb). Preziosi and colleagues found that the means of Hb at three months of age were the same (10.5 g/dL) among the 99 infants whose mothers received 100mg elemental Fe daily during pregnancy and the 98 infants whose mothers received placebo. Haemoglobin became lower (less favourable) in the supplemented (10.5 g/dL) than the placebo group (11.0 g/dL) at six months of age. Compliance was not reported in this study.

In Vietnam, the prevalence of anaemia is reported to be high (more than 43% [14] and 58% [15]) among pregnant women and 60% in infants aged less than 12 months [16]. IFA supplementation is recommended for women during pregnancy but not provided free of charge, including to women who are most economically disadvantaged [17]. Even if women can afford to purchase iron supplements, there are no legal requirements about the content of over-the-counter iron and folic acid supplements, leading to variety in quality and types of preparations. Health information for women about iron and nutrient rich foods is not widely available. There is no policy on iron fortification and iron-fortified food is not widely available in Vietnam.

The aim of this study was to examine the effect on infant Hb concentration and anaemia at six months of age of daily provision of antenatal iron—folic acid (IFA) supplementation, or twice weekly provision of antenatal IFA supplementation with or without other micronutrients, compared with the voluntary and self-financed approach that constitutes usual care in rural Vietnam.

## Methods

### Study design

The investigation was a secondary analysis of data generated in two larger studies conducted in Ha Nam Province, Vietnam. The first was a prospective population-based observational study (from 2009 to 2011, hereafter called the OS) which examined the effect of antenatal psycho-social factors, anaemia and iron deficiency in early and late pregnancy on infant development and health outcomes at 6 months of age. The second was a three-arm cluster randomised controlled trial of different antenatal iron supplement regimens including daily IFA, intermittent IFA, and intermittent MMN (from 2010 to 2012, hereafter called the CRT). The designs of the two studies have been reported in detail elsewhere [9,18].

### Setting

Both studies were conducted in Ha Nam, a rural province located in the north of Vietnam, approximately 50 km south of Hanoi. The population of Ha Nam is 0.8 million people with about 7.5% living below the international poverty line of USD1.25 per day. Almost all women have at least one antenatal health care visit and give birth in a medical facility. Iron supplements are not provided free for pregnant or non-pregnant women in Ha Nam.

### Participants

The participants of the OS were recruited in a two-stage sampling procedure. An independent statistician selected 50 of 116 communes in the province randomly. Then, all healthy women who were living in the selected communes and were less than 20 weeks pregnant with a single foetus during the enrollment period (December 2009 to January 2010) were eligible and invited to participate.

In the CRT, the eligibility criteria were being healthy and less than 16 weeks pregnant with a single foetus during the enrollment period (September to November 2010). Participants with severe anaemia (Hb < 8 g/dL) at recruitment were excluded. All rural communes of Ha Nam (104 communes) were included in the CRT and were allocated randomly to one of three arms: daily IFA (34 communes), twice weekly IFA (35 communes) and twice weekly MMN (35 communes). Randomisation was performed in Stata Version 9 (StataCorp LP, College Station, Texas, US) using ‘ralloc’ command by an independent statistician who was blinded to the identity of the communes. All eligible women living in these communes were invited to participate.

The original sample size of the OS was 497 and that of the CRT was > 400 per study arm. With approximately 20% loss of follow-up and 95% confidence, the above sample sizes have at least 95% statistical power to detect clinically important differences of 5 g/L for infant haemoglobin between any CRT arm and the OS (SD = 11g/L, ICC = 0.12).

### Interventions

The participants of the OS received standard care including free antenatal checks, giving birth in the commune health centre or the district or provincial hospitals and access to the National Growth Monitoring and Expanded Immunisation Programmes. In the CRT, women received IFA or MMN supplements in addition to standard care. The participants in arm 1 (daily IFA) received one tablet of IFA (60mg elemental iron and 0.4mg folic acid) taken daily; arm 2 (twice-weekly IFA), one capsule of IFA (60mg elemental iron and 1.5mg folic acid) taken twice per week; and arm 3 (MMN) one capsule of MMN (60 mg elemental iron, 1.5 mg folic acid per capsule; plus a variation of the dose of the micronutrients in the UNIMMAP supplement [5]) taken twice per week. The participants in the CRT were visited every 6 weeks at home from recruitment to provide the supplements for each subsequent 6 week period. Participants and the research teams who were involved in fieldwork and laboratory analyses were blinded to the intervention arms. Information about compliance of the participants with the regimens was collected during the 6-weekly visits. Detailed information on the interventions in the CRT have been reported elsewhere [18].

### Data sources

Data for the two studies were collected by the same data collection team from the Hanoi Research and Training Centre for Community Development (RTCCD). Data were collected in four waves in both studies: the first (Baseline) was at the time of recruitment; the second (Follow-up 1) when participants were at least 28 weeks pregnant; the third (Follow-up 2) 6–8 weeks after birth; and the last (Follow-up 3) 6 months after birth.

#### Baseline data

Baseline data including socio-demographic characteristics and women’s reproductive health were collected by study-specific questions, which have been used in previous studies in the same settings and found to be comprehensible to rural women and to yield interpretable data [19,20]. Data collected included women’s age, highest education level, occupation, marital status, and reproductive history including gravidity, parity, spontaneous abortions and foetal or neonatal deaths.

In both the OS and the CRT, maternal Hb was evaluated at Baseline from a finger prick blood sample, using a hemoglobinometer (HemoCue AB, Angelholm Sweden) and a sample of venous blood was taken and centrifuged. Serum was frozen at -20C and transported to Australia on dry ice for ferritin analysis. Serum ferritin was analysed by Chemiluminescent Microparticle Immuno Assay performed on the Archicentre ci62000 instrument (Abbott, Illinois, USA) at Alfred Pathology Services, Alfred Health, Australia.

#### Infant data

In this study, the main outcome was infant Hb concentration measured at Follow-up 3 (6 months of age). Infant Hb was evaluated using a hemoglobinometer (HemoCue AB, Angelholm Sweden) in the field from heel prick sample of blood. Infants were classified as having anaemia when Hb < 11.0 g/dL [21].

### Data analysis

The baseline characteristics were compared among the four groups of women: the group who participated in the OS and the groups who comprised the three arms of the CRT. The baseline characteristics among these four groups were presented as means (SD), medians (25^th^-75^th^ percentile) or frequency (per cent), and compared using one-way ANOVA, nonparametric k-sample test on the equality of medians, chi-square test, and Fisher's exact test, where appropriate.

The mean differences in infant Hb of the four groups were calculated by a multivariable linear regression model that included all baseline characteristics that were significantly different among the groups. The odds ratios of infant anaemia between each of the three arms and the observational study were calculated by a multivariable logistic regression model that also included all baseline characteristics that were significantly different among the groups. Cluster effect due to commune was controlled in these models using the Huber-White Sandwich estimator.

The inclusion criteria were slightly different between the OS and the CRT in that women with severe anaemia at baseline and those living in urban communes were not included in the CRT but were included in the OS. Sensitivity analyses were carried out that excluded women who were severely anaemic at baseline and who were from urban communes to verify the main findings.

Analyses followed intention-to-treat principles and were performed using Stata, Version 12 (StataCorp, TX, USA). Only women whose infant’s Hb was assessed at Follow-up 3 were included in analyses.

### Ethics approvals

Approvals to conduct the OS were provided by the Ha Nam Provincial Health Department Ethics Committee, the Vietnam Medical Association Ethics and Scientific Committee and the University of Melbourne’s Health Sciences Human Research Ethics Committee. The study protocol of the CRT was approved by the Melbourne Health Human Research Ethics Committee, and the Hanam Provincial Human Research Ethics Committee and registered in Australian New Zealand Clinical Trials (Registry: ANZCTR 12610000944033). Permission to transport biological samples from Vietnam to Australia for analyses was provided by the Vietnam Ministry of Health.

## Results

### Sample

**Fig 1:**
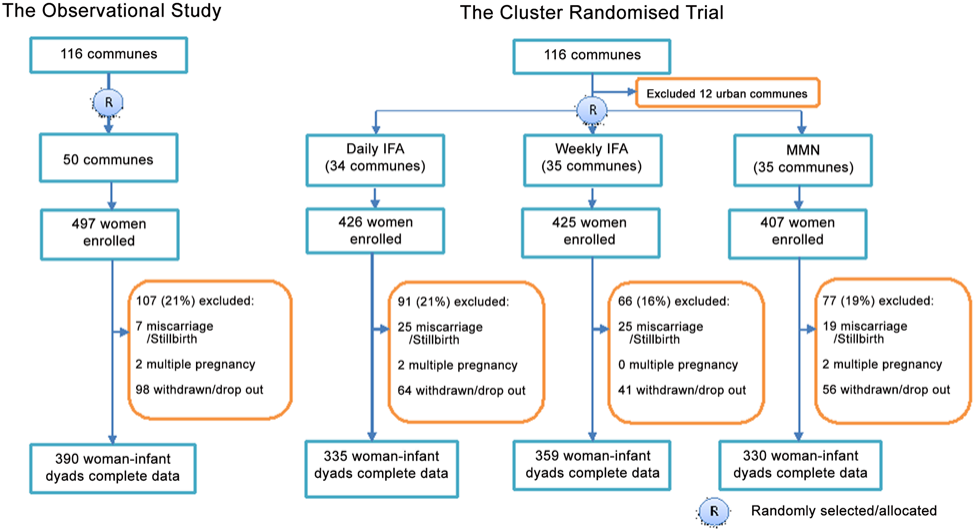
Numbers of participants and attrition by studies.

The numbers of women enrolled in the two studies and the numbers of pairs of women and infants included (provided complete data) and excluded (missing follow-up data) are presented in Fig 1. The baseline characteristics of women who were and who were not included in the analyses are described in S1 Table. No significant differences were found.

The baseline characteristics of women who were included in the analyses among the four groups of women (OS and three arms of the CRT) are shown in Table 1. The distributions of age, education level, and occupation were similar for the four groups. However, parity, gestational age at baseline survey, Hb, anaemia, and ferritin were significantly different between at least one group and the others. The proportion of nulliparous women was lowest in weekly IFA group and highest in daily IFA group. Mean gestational age at baseline was about four weeks higher in the OS group than the CRT groups and is the probable explanation for the differences in Hb and ferritin at baseline between the participants of the two studies (S1 and S2 Figs).

**Table 1:** Baseline characteristics of the participants.

Characteristic	Observational study (N = 389)	Daily IFA (N = 335)	Weekly IFA (N = 359)	MMN (N = 330)	p-value^(2)^
Age (years), mean (SD)	26.1 (4.9)	25.0 (4.8)	26.1 (4.7)	25.9 (4.9)	0.85
Highest education, No. (%)					0.11
Partial or complete primary school (Grades 1–5)	72 (18.5)	57 (17.0)	42 (11.7)	57 (17.3)	
Secondary school (Grades 6–9)	210 (53.9)	162 (48.4)	199 (55.4)	162 (49.1)	
High school (Grades 10–12)	48 (12.3)	53 (15.8)	52 (14.5)	59 (17.9)	
Any post-secondary education	60 (15.4)	63 (18.8)	66 (18.4)	52 (15.8)	
Occupation, No. (%)					0.40
Farmer	176 (50.1)	139 (41.5)	153 (42.6)	139 (42.1)	
Factory, handcraft worker or retailer	123 (31.5)	111 (33.1)	123 (34.3)	105 (31.8)	
Government or private officer	47 (12.1)	44 (13.1)	53 (14.8)	37 (11.2)	
Not currently engaged in income-generating activity	44 (11.3)	41 (12.2)	30 (8.4)	49 (14.9)	
Nulliparous, No. (%)	129 (33.1)	123 (36.7)	91 (25.4)	106 (32.1)	0.01
Gestational age at baseline survey (weeks), mean (SD)	16.7 (3.0)	12.4 (3.3)	12.1 (3.2)	12.1 (3.5)	0.01
Haemoglobin concentration (g/dL), mean (SD)	11.8 (1.2)	12.5 (1.4)	12.1 (1.1)	12.3 (1.2)	<0.001
Anaemia^(1)^, No. (%)	85 (21.8)	40 (12.0)	52 (14.5)	44 (13.3)	0.001
Ferritin (ng/ml), median (IQR)	41.2 (22.3;71.7)	74.5 (48.0;130)	78 (51.0;128)	78 (49.0;126)	<0.001

^(1)^Anaemia defined as haemoglobin < 11 g/dL;

^(2)^Significant tests include one-way ANOVA, nonparametric k-sample test on the equality of medians, and chi-square test, where appropriate.

### Compliance

Compliance with the regimens in the CRT is reported in detail elsewhere [18]. The median compliance level (number of supplements consumed/number of supplements provided) was 91% in the daily IFA group, 96% in the weekly IFA group, and 85% in the MMN group. In the OS group, 10.3% of women did not take any iron supplements during pregnancy. For the 89.7% of women who did take supplements the median duration of pregnancy (%) was 59.7% (IQR of 35.5% to 76.1%, range 2.8% to 100%) [22].

### Infant haemoglobin and anaemia

**Fig 2:**
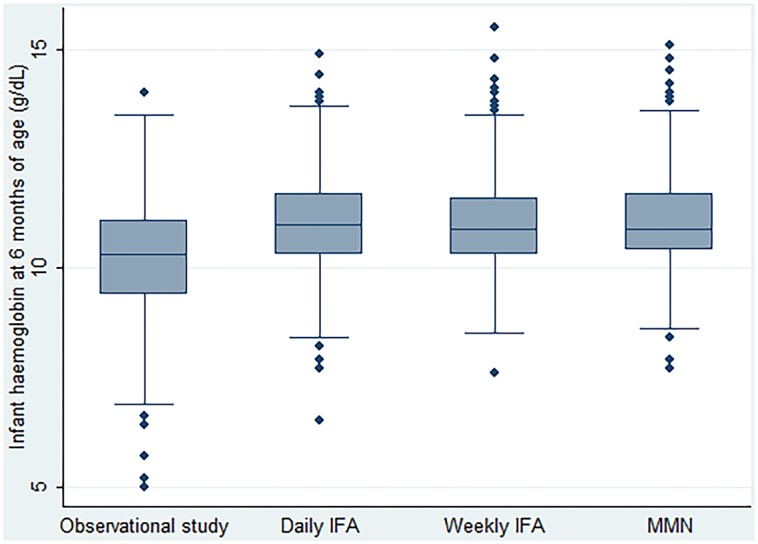
Distributions of infant haemoglobin at 6 months of age by studies.

The descriptive analyses show that the distribution of infant Hb in the OS group was lower than in the three groups of the CRT (Table 2 and Fig 2). The prevalence of anaemia was higher in infants in the OS group than infants in the three groups of the CRT (Table 2). The differences of mean Hb and prevalence of anaemia among the three groups of the CRT were small.

**Table 2:** Characteristics of infants.

Characteristic	Observational study (N = 389)	Daily IFA (N = 335)	Weekly IFA (N = 359)	MMN (N = 330)	p-value^(2)^
Infant sex (boy), No. (%)	177 (45.4)	164 (48.9)	160 (44.6)	165 (50.0)	0.38
Birthweight (kg), mean (SD)	3.10 (0.46)	3.14 (0.41)	3.18 (0.36)	3.13 (0.41)	<0.001
Haemoglobin at 6 months of age (g/dL), mean (SD)	10.22 (1.4)	11.03 (1.1)	10.98 (1.1)	11.11(1.2)	<0.001
Anaemia^(1)^ at 6 months of age, No. (%)	267 (68.6)	158 (47.2)	192 (53.5)	166 (50.3)	<0.001

^(1)^Anaemia defined as haemoglobin < 11 g/dL;

^(2)^Significant tests include one-way ANOVA, nonparametric k-sample test on the equality of medians, and chi-square test, where appropriate.

The trends observed in the descriptive analyses were confirmed in regression analyses when adjusted for infant sex and the baseline characteristics: maternal ferritin, haemoglobin concentration, parity and gestational age (Table 3). The mean infant Hb in the CRT groups was significantly higher by an estimated mean of 1 g/dL than in the OS. The odds of infants in the CRT being anaemic were significantly lower by approximately 0.3 fold than those in the OS. There were no significant differences in mean Hb or odds of infant anaemia between the three Trial groups.

**Table 3:** Estimated mean difference of infant haemoglobin and odds ratios of infant anaemia at 6 months of age between three intervention groups and the observational group.

Study group	Infant haemoglobin (g/dL) ^(1)^		Infant anaemia^(2)^	
	Mean difference (95% CI)	p-value	odds ratio (95% CI)	p-value
Observational study	Ref.		Ref.	
Daily IFA	.95 (.71 to 1.18)	< 0.001	.31 (.22 to .43)	< 0.001
Weekly IFA	.91 (.69 to 1.12)	< 0.001	.38 (.26 to .54)	< 0.001
Weekly MMN	1.04 (.80 to 1.27)	< 0.001	.33 (.23 to .48)	< 0.001

CI—Confidence Interval; IFA—Antenatal iron-folic acid supplementation; MMN—Antenatal multiple micronutrient supplementation

^(1)^Multiple linear regression model adjusted for infant sex, and baseline data including maternal ferritin (log transformed), haemoglobin concentration, parity and gestational age at baseline, and allowing for the effect of clustering using robust standard errors.

^(2)^Multiple logistic regression model adjusted for infant sex, and baseline data including maternal ferritin (log transformed), haemoglobin concentration, parity, and gestational age at baseline, and allowing for the effect of clustering using robust standard errors.

The two models presented in Table 3 were verified in sensitivity analyses that excluded women in the OS who were severely anaemic at baseline (2 women) and who were from urban communes (35 women). The results in Table 3 did not change materially in the sensitivity analyses.

## Discussion

This is the first study to compare the impact of daily antenatal IFA, twice weekly provision of antenatal IFA (with or without other micronutrients) to usual voluntary and self-financed use of iron on infant haemoglobin and anaemia in the world. Our data demonstrate that that the effects on infant Hb and anaemia of the three supplementation regimens were not significantly different and that all were statistically and clinically superior to voluntary and self-financed usual care. Despite the benefit of supplementation the prevalence of anaemia was extremely high in infants in all groups in this study.

This study analysed data from two original studies that together provided sufficient mother-infant dyads (1413 pairs of women and infants) to compare the impact of different supplementation regimens with usual care. The effect sizes of the intervention conditions on infant Hb and anaemia were calculated by multivariable models that took into account clustering by village and baseline characteristics in which there were significant differences among the study groups. We acknowledge that using data from two different studies could introduce bias in the results. We believe that this source of bias was limited because the methods of the two studies were almost identical, the two studies were conducted by the same research team, and were conducted very close together in that the two recruitment phases were only 10 months apart. Relatively high attrition rates in this study (21% in the daily IFA and the OS groups, 19% in the MMN and 16% in the weekly IFA) could affect the findings in either direction. However, we compared the baseline characteristics of the women who did and did not provide complete data and the distributions were similar. There were several differences in the baseline characteristics among the participants of the two studies including Hb and ferritin concentrations. The final results have controlled for these differences through multiple linear and logistic regression models.

This study did not include a placebo group. It is not ethical to give placebo to pregnant women in low- and middle-income countries where antenatal iron supplementation is recommended. This study used the participants of an observational study as the comparison group that allowed us to determine the effects of different antenatal iron supplementation interventions compared to no intervention. The results are informative to public health policy makers indicating that they should consider providing iron supplementation to pregnant women free of charge to improve child health outcomes instead of just recommending to women that they purchase and use antenatal iron supplementation.

The mean infant Hb at 6 months of age and the comparisons of this factor among the three arms of the CRT have been reported elsewhere [18]. The present study reported the mean differences of infant Hb between each CRT arm and the observational study using multiple regression analyses taken into account a wide range of baseline characteristics. The main findings of this study are novel and elucidate the implications for public health strategies.

Our data demonstrate that in this rural province in a lower-middle income country antenatal iron supplements had sustained benefits for infant Hb anaemia that were still detectable at 6 months of age. These are in contrast to the findings of Preziosi et al.’s trial in Niger [13] that did not find a beneficial effect of daily antenatal iron supplementation on infant Hb at six months of age. There were several factors that might have influenced the results in Preziosi et al.’s trial. First, baseline differences in the characteristics of the two arms including age (significantly younger in the iron group) and parity (significantly higher in the iron group) were not taken into account. Second, the compliance rates were not reported. A low rate of compliance to the regimen could alter the effect of the intervention in the whole sample. Third, the intervention was started late in pregnancy (at a mean of 28 gestational weeks) which reduced the period during which iron stores could be increased in the mother and be available to the growing foetus. Finally, the relatively small sample size (99 women in the iron supplemented group and 98 in the placebo group) of this trial may not have been enough to control for the effect of other common causes of anaemia such as malaria, helminth infection, and deficiencies of vitamin B12 and folic acid that could differ between women receiving iron supplementation and women receiving placebo.

Our data confirm that the effects on infant Hb and anaemia of daily IFA and intermittent IFA supplementation during pregnancy were not significantly different. This finding is consistent with existing evidence about the effects of these regimens on birth outcomes, maternal health outcomes, and infant development [18,23]. Therefore, intermittent antenatal IFA supplementation with the potential improvements in compliance and lower costs to individuals or the health system should be considered in public health policies to promote optimal infant health in low- and lower-middle-income countries where anaemia rates are low, or where a weekly iron-folic acid supplementation (WIFS) program for women of reproductive age has been in place for 12 months or more.

Intermittent MMN (IFA supplementation in combination with other micronutrients) in pregnancy had similar effects on infant Hb and anaemia as daily IFA and intermittent IFA alone. However, MMN was found to have more side effects (nausea and vomiting) and lower adherence in this study [18]. Previous trials have found additional beneficial effects of antenatal intermittent MMN compared to IFA regimes on birthweight but not on other pregnancy outcomes [7] or child growth ([8]. Further studies are warranted to identify the cost-effectiveness and safety of antenatal intermittent MMN.

In conclusion, infant anaemia is a public health problem in Vietnam and in other resource-constrained nations. Provision of antenatal daily IFA, twice weekly IFA, or twice weekly MMN may have clinically significantly positive effect on infant Hb and reduces the risk of anaemia in infants. Universal provision of these intermittent iron supplements free of charge should be considered in Vietnam as well as other low- and lower-middle-income countries, where the prevalence of anaemia among women is known to be low.

## Supporting Information

S1 Fig Women’s haemoglobin (g/dL) by gestational ages (weeks) at baseline. (TIF)

S2 Fig Women’s ferritin (g/dL) by gestational ages (weeks) at baseline. (TIF)

S1 Table Baseline characteristics of women who were included in and excluded from the analyses. (DOCX)
