# Supplementary figures and images for: Antenatal Iron Supplementation Regimens for Pregnant Women in Rural Vietnam and Subsequent Haemoglobin Concentration and Anaemia among Their Infants
Source: PLoS One. 2015 Apr 30;10(4):e0125740. doi: 10.1371/journal.pone.0125740 (PMC4416008; doi:10.1371/journal.pone.0125740)

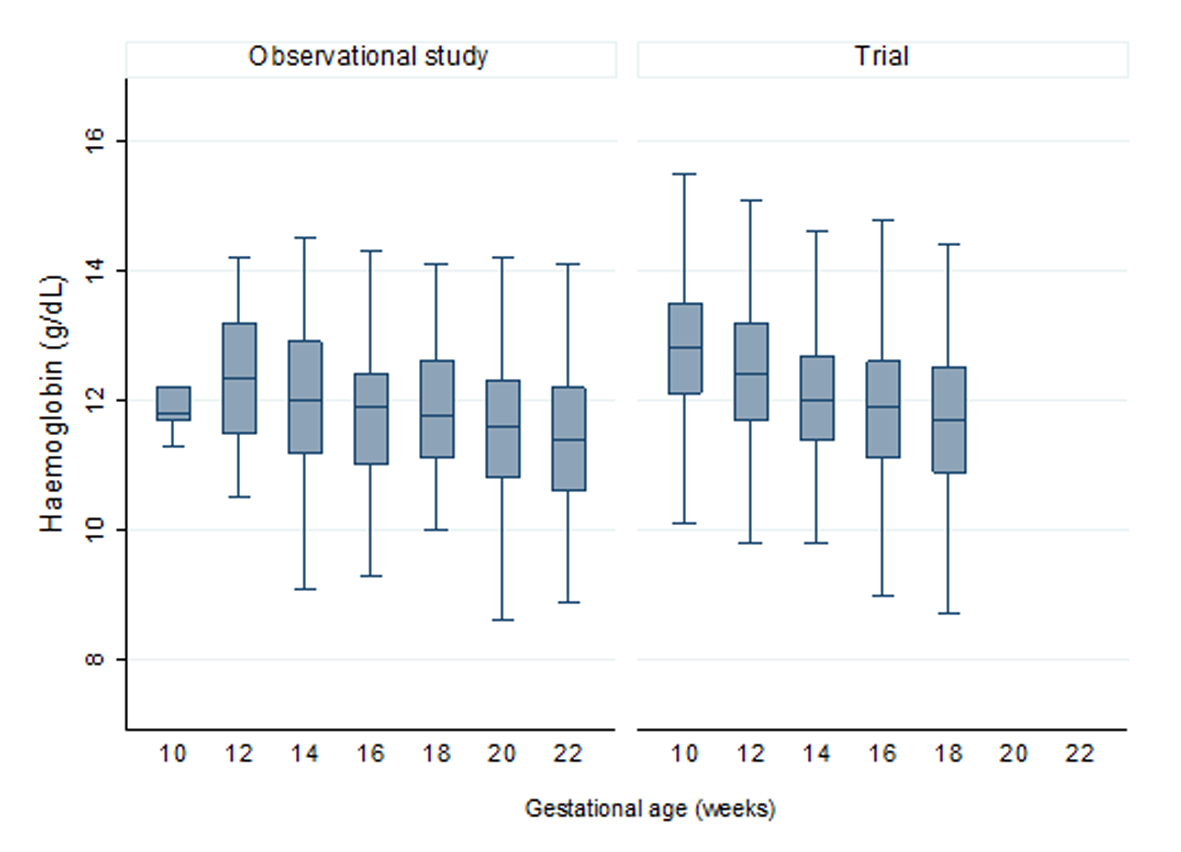

Supplement: S1 Fig — (TIF) [file pone.0125740.s001.tif]

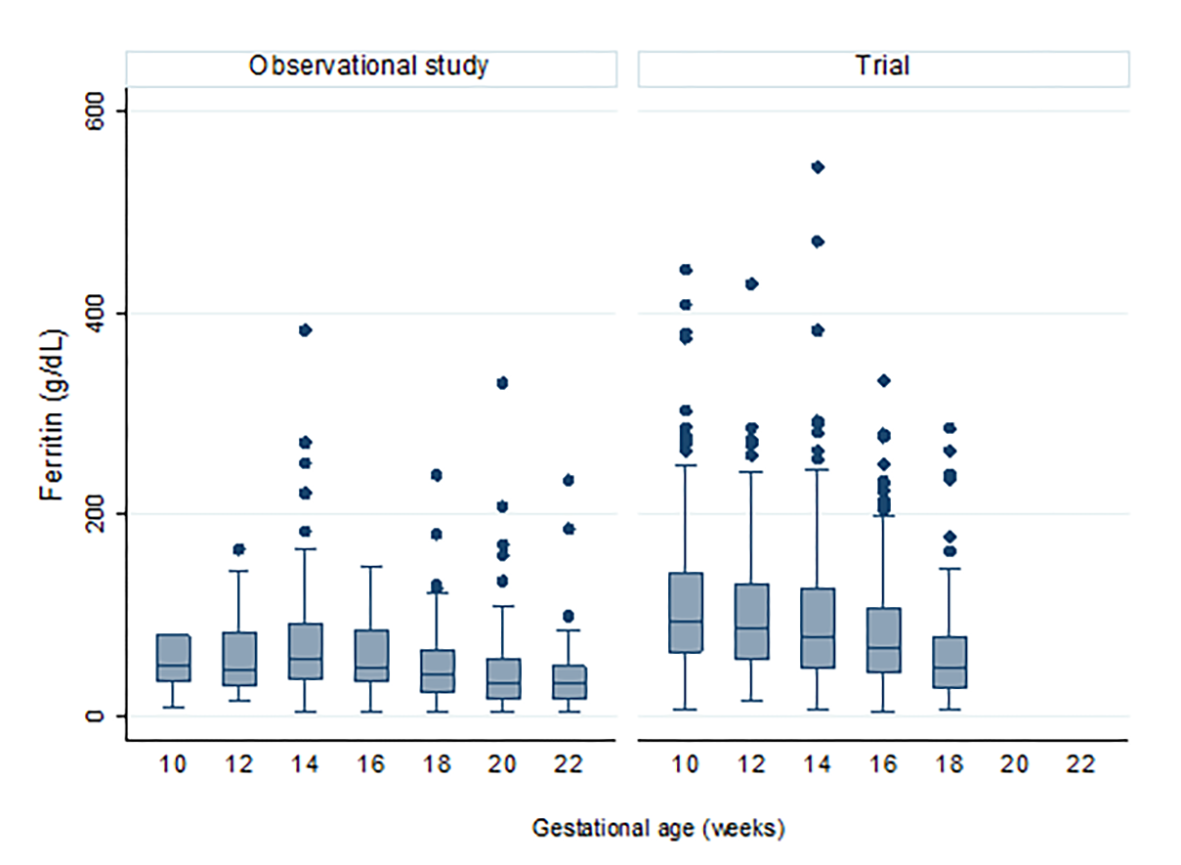

Supplement: S2 Fig — (TIF) [file pone.0125740.s002.tif]
