# Supplementary material for: Antenatal Iron Supplementation Regimens for Pregnant Women in Rural Vietnam and Subsequent Haemoglobin Concentration and Anaemia among Their Infants
Source: PLoS One. 2015 Apr 30;10(4):e0125740. doi: 10.1371/journal.pone.0125740 (PMC4416008; doi:10.1371/journal.pone.0125740)
Supplement: S1 Table — (DOCX) [file pone.0125740.s003.docx]

| Characteristic | Observational study | | The Cluster Randomised Trial | | | | | |
| --- | --- | --- | --- | --- | --- | --- | --- | --- |
|  |  |  | Daily IFA | | Weekly IFA | | MMN | |
|  | Included (N=390) | Excluded (N=108) | Included (N=335) | Excluded (N=91) | Included (N=359) | Excluded (N=66) | Included (N=330) | Excluded (N=77) |
| Age (years), mean [SD] | 26.1 [4.9] | 25.8 [4.5] | 25.0 [4.8] | 26.1 [5.9] | 26.1 [4.7] | 26.7 [4.6] | 25.9 [4.9] | 26.2 [5.4] |
| Highest education, No. (%) |  |  |  |  |  |  |  |  |
| Partial or complete primary school (Grades 1–5) | 72 (18.5) | 21 (19.6) | 57 (17.0) | 21 (23.1) | 42 (11.7) | 12 (18.2) | 57 (17.3) | 9 (11.7) |
| Secondary school (Grades 6–9) | 210 (53.9) | 49 (45.8) | 162 (48.4) | 38 (41.8) | 199 (55.4) | 30 (45.6) | 162 (49.1) | 37 (48.1) |
| High school (Grades 10–12) | 48 (12.3) | 10 (9.4) | 53 (15.8) | 14 (15.3) | 52 (14.5) | 9 (13.6) | 59 (17.9) | 6 (7.8) |
| Any post-secondary education | 60 (15.4) | 27 (25.2) | 63 (18.8) | 18 (19.8) | 66 (18.4) | 15 (22.7) | 52 (15.8) | 25 (32.5) |
| Occupation, No. (%) |  |  |  |  |  |  |  |  |
| Farmer | 176 (50.1) | 46 (43.0) | 139 (41.5) | 32 (35.2) | 153 (42.6) | 27 (40.9) | 139 (42.1) | 23 (29.9) |
| Factory, handcraft worker or retailer | 123 (31.5) | 35 (32.7) | 111 (33.1) | 33 (36.3) | 123 (34.3) | 22 (33.3) | 105 (31.8) | 25 (32.5) |
| Government or private officer | 47 (12.1) | 14 (13.1) | 44 (13.1) | 17 (18.7) | 53 (14.8) | 13 (19.7) | 37 (11.2) | 22 (28.6) |
| Not currently engaged in income-generating activity | 44 (11.3) | 12 (11.2) | 41 (12.2) | 9 (9.9) | 30 (8.4) | 4 (6.1) | 49 (14.9) | 7 (9.1) |
| Nulliparous, No. (%) | 129 (33.1) | 43 (40.1) | 123 (36.7) | 25 (27.5) | 91 (25.4) | 16 (24.2) | 106 (32.1) | 24 (31.1) |
| Haemoglobin concentration (g/dL), mean [SD] | 11.8 [1.2] | 11.5 [1.1] | 12.5 [1.4] | 12.6 [1.4] | 12.1 [1.1] | 12.5 [1.0] | 12.3 [1.2] | 12.5 [1.2] |
| Anaemia^(1)^, No. (%) | 85 (21.8) | 27 (25.2) | 40 (12.0) | 9 (10.0) | 52 (14.5) | 5 (7.6) | 44 (13.3) | 9 (11.7) |
| Ferritin (ng/ml), median {IQR} | 41.2 {22.3;71.7} | 48.8 {29.8;71.6} | 74.5 {48.0;130} | 79 {45;11.8} | 78 {51.0;128} | 92.5 {61;145.0} | 78 {49.0;126} | 89 {56.0;124} |

^(1)^ - Anaemia defined as haemoglobin < 11 g/dL
